# Robotic Conversion of Single Anastomosis Duodeno-Ileal Sleeve (SADI-S) to Roux-en-Y Gastric Bypass (RYGB): A Novel Approach

**DOI:** 10.7759/cureus.104840

**Published:** 2026-03-07

**Authors:** Ahmad E Al-Mulla, Abdulla Sultan, Giorgio A Barretta, Maher Elchaar

**Affiliations:** 1 Surgery, Farwaniya Hospital, Farwaniya, KWT; 2 Surgery, Nossa Senhora das Graças, Curitiba, BRA; 3 Surgery, St. Luke’s University Hospital, Bethlehem, USA

**Keywords:** bariatric revision, gastric bypass, malnutrition, robotic surgery, roux-en-y, sadi-s

## Abstract

The single anastomosis duodeno-ileal sleeve (SADI-S) presents a simplified alternative to the duodenal switch. However, this procedure may result in malabsorption and associated complications for some patients. Revisional bariatric surgery can be extremely challenging, particularly following a previous intervention, and conventional laparoscopy may be limited in these complex cases. The introduction of robotic surgery has improved both precision and safety in such cases. This report describes a scarce case of robotic conversion from SADI-S to Roux-en-Y gastric bypass, illustrating its feasibility and favorable patient outcomes.

## Introduction

The global obesity epidemic has driven the evaluation of bariatric surgery, with procedures shifting from restrictive operations to combined techniques incorporating malabsorption. Single-anastomosis duodeno-ileal sleeve (SADI-S) can be considered either a primary or a second-stage procedure, often performed after sleeve gastrectomy (SG) [1]. Despite the advantages of SADI-S, it is associated with complications such as malabsorption, weight regain, vitamin deficiency, and marginal ulcers, thus requiring revisional surgery [2]. Conversion to Roux-en-Y gastric bypass (RYGB) is often performed to reduce malabsorption, restore regular alimentary continuity, and aid weight loss. However, revisional surgery is often challenging and highly demanding, particularly when performed laparoscopically due to limited freedom in small spaces, 2D visualization, spatial disorientation, ergonomic difficulties, and difficult camera movement, leading to a high conversion rate [3].

Robotic surgery is becoming increasingly common in bariatric procedures, especially in revision surgeries, where dense adhesions and altered anatomy pose significant challenges [4]. The advantages of robotic surgery, such as 3D vision, enhanced wrist mobility, tremor reduction, and a surgeon-controlled camera, help overcome the limitations associated with traditional laparoscopic techniques [5]. In this report, we present a rare case of converting from a previous SADI-S to a RYGB in a patient who experienced prolonged hypocalcemia.

## Case presentation

This case involves a 33-year-old female patient who was admitted through our outpatient department. She has a history of undergoing an SG in 2010 due to a BMI of 51 kg/m². Over the course of 10 years, she lost approximately 31 kg but subsequently regained more than 50% of that weight. In 2022, she underwent an SADI-S procedure as a revisional surgery, with resizing of the sleeve and the creation of a 270-cm common limb. After this second procedure, she experienced significant weight loss (BMI: 25 kg/m^2^) and maintained good nutritional status. However, during her recent follow-ups, she reported frequent bowel movements and episodes of dizziness that began six months before her admission. She had also been hospitalized previously for lethargy, dizziness, and frequent bowel movements. During that time, she was managed conservatively, advised to consult a dietitian, adhered to her supplement regimen, and followed up at our bariatric outpatient department.

In September 2025, she was readmitted, complaining of frequent bowel movements, vomiting, and generalized weakness. Upon examination, she was vitally stable but exhibited some physical abnormalities. Laboratory tests revealed decreased levels of protein (40 g/L), albumin (28 g/L), and vitamin D (22 ng/mL). Other investigations were unremarkable, including vitamin B_6_ and B_12_. Notably, she presented with persistent hypocalcemia (1.52-1.9 mmol/L) and elevated parathyroid hormone (28.9 pmol/L) levels (Table 1), despite efforts by her treating team and an endocrinologist to correct this condition. Further investigations, including sestamibi scans and neck ultrasounds, were performed to address her hypocalcemia. Additional investigations, such as FibroScan, abdominal ultrasound, endoscopy, and colonoscopy, revealed unremarkable findings.

**Table 1:** Pre-operative and post-operative investigations

Parameter	Pre-revision value	Reference range (adult female)	Post-revision value	Interpretation
Total protein (g/L)	40	60-80 g/L	—	Hypoproteinemia
Albumin (g/L)	28	35-50 g/L	—	Hypoalbuminemia
Vitamin D, 25(OH) (ng/mL)	22	30-100 ng/mL	—	Insufficiency
Serum calcium (mmol/L)	1.52-1.9	2.1-2.6 mmol/L	2.2-2.35	Normalized post-revision
Parathyroid hormone (pmol/L)	28.9	1.6-6.9 pmol/L	—	Secondary hyperparathyroidism

The patient was initially on a clear liquid diet, which later progressed to a regular oral diet. She received vitamins and calcium supplementation through intravenous infusion; however, we were unable to maintain her calcium levels within the normal range. Additionally, she continued to experience frequent bowel movements, reporting up to 10 times a day. As a result, we decided to revise her surgical procedure to a less malabsorptive option, such as RYGB. Conceding the former bariatric history of the patient, we decided to consider a robotic revisional approach. The patient consented to the robotic surgery and was prepared for the procedure following a consultation with the anesthesiology team and signing of the consent form.

**Figure 1:**
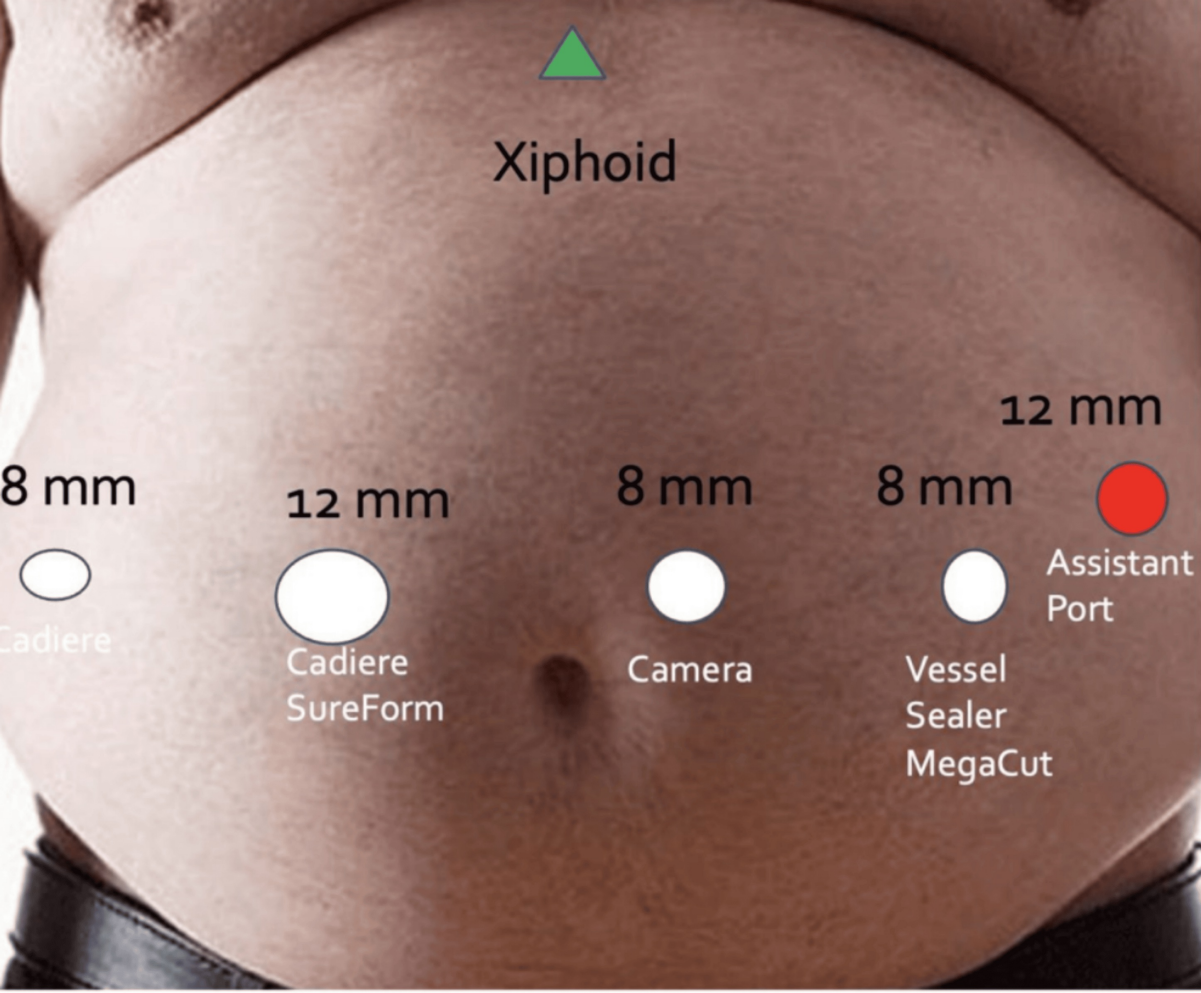
Port locations for upper GI robotic surgery

The port placement was configured in the upper abdomen, with five ports arranged along a horizontal line across the abdomen (Figure 1). Liver retraction was performed just below the xiphoid process. The procedure began with the dissection of adhesions from previous surgeries. Initially, a laparoscopic approach was used to measure the common limb length, which, surprisingly, measured 217 cm. Afterward, the robot was docked, and further dissection of the previous procedure site was performed.

**Figure 2:**
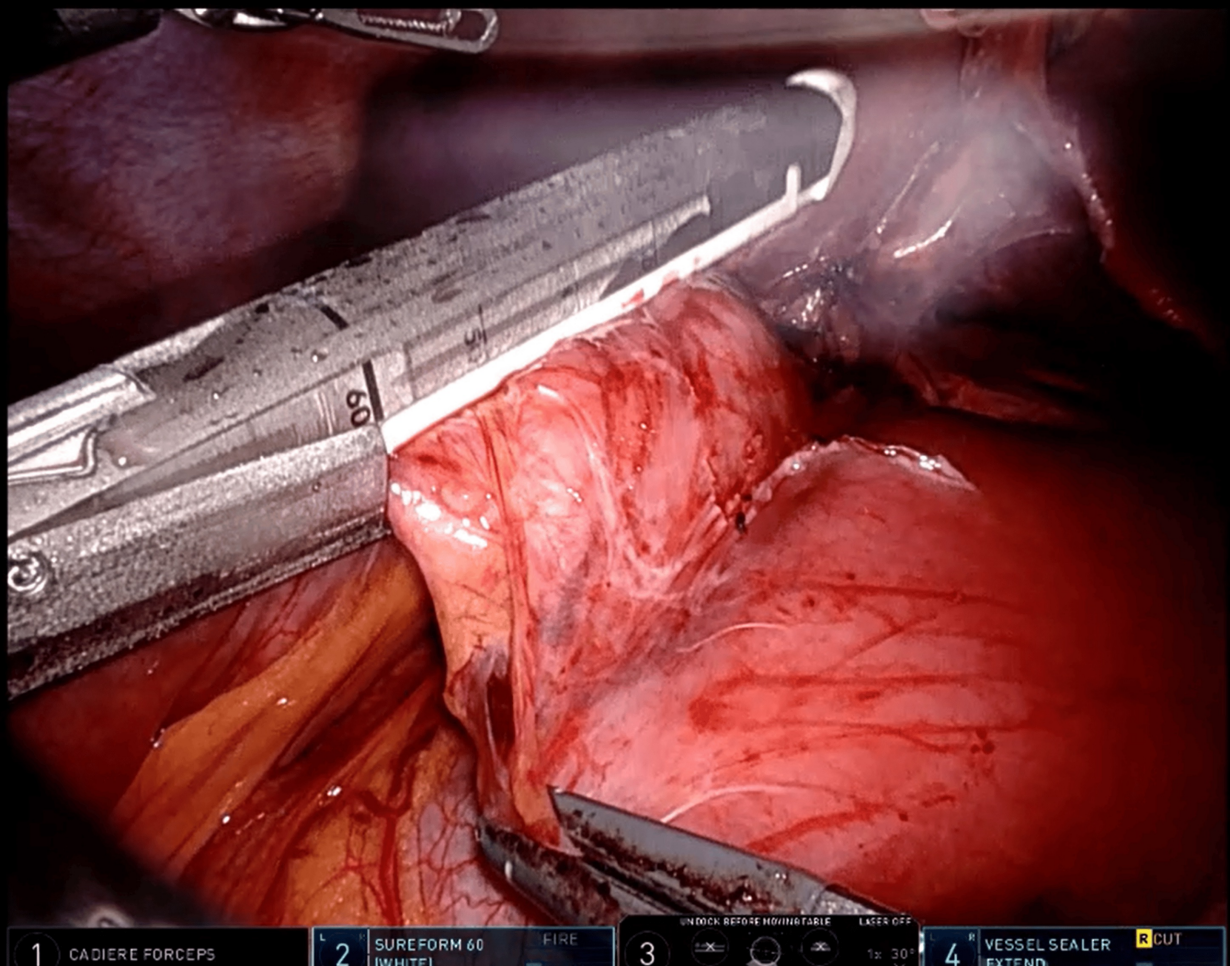
Duodeno-ileal anastomosis divided using a white cartridge stapler

**Figure 3:**
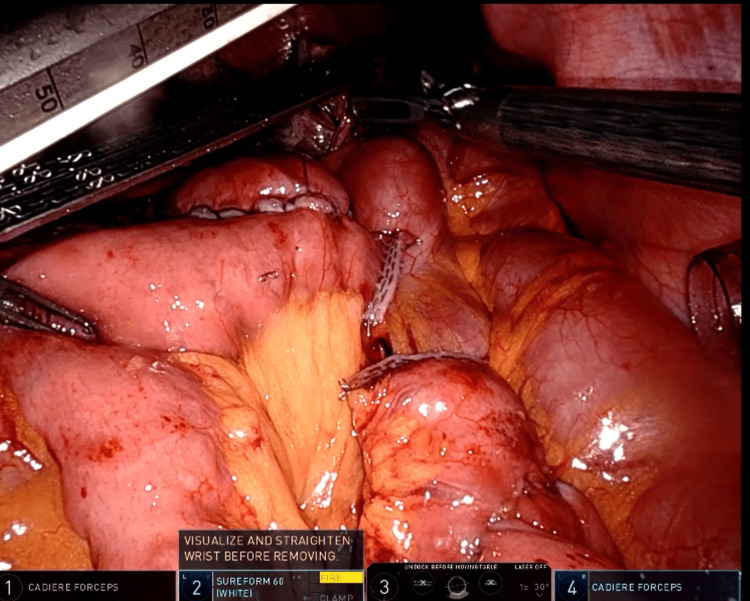
Roux-en-Y gastric bypass with 70 cm biliopancreatic limb and 70 cm alimentary limb

The first step involved dividing the previous duodeno-ileal anastomosis using a white cartridge (Figure 2). Next, the stomach was divided, and a small pouch was created for the gastro-jejunal anastomosis using a blue cartridge. The biliopancreatic limb was constructed to be 70 cm from the Treitz ligament, while the alimentary limb was constructed to a length of 70 cm. All enterotomy openings were closed with barbed (V-Loc 3-0) sutures, potential spaces for internal hernia were closed with non-absorbable (Ethibond 2-0) sutures, and the biliopancreatic limb was divided using a white cartridge (Figure 3). The procedure lasted approximately three hours.

The patient's post-operative course was unremarkable. Fluids were initiated on the same day, and she was encouraged to mobilize immediately upon full recovery. Calcium levels normalized (2.2-2.35 mmol/L) (Table 1) and remained stable with supplementation. The patient was discharged two days postoperatively and was advised to follow up in our outpatient department.

During her first follow-up visit after two weeks, the patient experienced mild nausea and vomiting and was reassured. All investigations were normal, showing no decrease in calcium levels (2.25 mmol/L), and she continued to be followed closely (Table 1).

## Discussion

SADI-S was first described in 2007 and is known for achieving significant weight loss with a lower complication rate [6]. It accounts for 2% of metabolic bariatric surgery (MBS) procedures performed in the US [7]. Despite being considered a relatively safe procedure, it requires close patient follow-up to prevent potentially life-threatening complications. Some complications reported in the literature include marginal ulcers, bile reflux, duodenal stump blowout, and malnutrition [8].

Malnutrition is one of the most concerning long-term complications associated with bariatric surgery. It has been linked to malabsorptive procedures such as jejunoileal bypass, biliopancreatic diversion, and duodenal switch (DS). While SADI-S is an alternative, milder, and more straightforward malabsorptive procedure compared to the DS, it can still lead to malnutrition in some patients, especially if they have a shorter common channel (<250 cm) [9]. This was discussed in our previous case report; the patient required revision surgery after failing to progress and maintain their nutritional status.

Revisions in bariatric surgery are generally more complex than primary surgeries. Factors such as adhesions and altered anatomy contribute to an increased risk of blood loss, longer operative times, extended hospital stays, admission to the intensive care unit, post-operative complications, and early readmissions to the hospital [10]. Approximately 28% of patients undergoing MBS may require revision. In 2019, 16.8% of MBS patients underwent revisional surgery [11].

Robotic revisional bariatric surgery (R-RBS) is gaining popularity, especially in cases where precise suturing, detailed dissection, and enhanced visualization are crucial. Studies show that R-RBS results in better outcomes and shorter hospital stays than traditional laparoscopic surgery [12,13]. This success can be attributed to the standardization of techniques in practice and in the operating theater, which leads to shorter operative times and higher turnover rates [14].

In our case report, we chose the RYGB procedure, which offers a balance between restriction and malabsorption while minimizing the risks of severe protein deficiency and bile reflux. These complications may arise with other surgical options. RYGB was deemed a better choice than revising the common limb, as re-anastomosing the duodenum can be technically challenging [1].

The use of R-RBS in our case represents a novel approach and has not been described in the literature to date. While there have been instances of converting a SADI-S to either RYGB or one anastomosis gastric bypass, these procedures were performed laparoscopically [1,15]. Thus, this specific conversion is rare and has not been documented in the literature, underscoring the significance of our case report.

## Conclusions

Revisional robotic bariatric surgeries are becoming increasingly popular due to their high precision and low complication rates. Many complex cases have been completed using robotic surgery, which can be challenging with traditional laparoscopy. Converting from SADI-S to RYGB is feasible and can be safely performed in experienced centers. This case contributes to the limited literature on the subject and highlights the potential of robotic platforms in managing severe complications.
